# A hepatitis B virus RNA-sensing and RNA-editing-dependent reporter system

**DOI:** 10.1128/jvi.00922-25

**Published:** 2025-10-10

**Authors:** Liren Sun, Andrew Snedeker, Liudi Tang

**Affiliations:** 1 Department of Experimental Therapeutics, Baruch S. Blumberg Institute 480639, Doylestown, Pennsylvania, USA; Wake Forest University School of Medicine, Winston-Salem, North Carolina, USA

**Keywords:** HBV, reporter, RADARS, RNA editing

## Abstract

The compact genomic organization of hepatitis B virus (HBV) has long hindered the development of reporter viruses that do not compromise viral gene expression and replication. Leveraging the advantage of an RNA-sensing and ADAR-editing-dependent cellular reporter system termed reprogrammable adenosine deaminase acting on RNA sensors (RADARS), we developed an HBV-RADARS reporter. In this system, the expression of the reporter gene is activated *in trans* by HBV RNA-guided, cellular adenosine deaminase acting on RNA 1 (ADAR1)-dependent reporter RNA editing. Using a luciferase reporter, we systematically scanned all ADAR1 targetable sites present in HBV RNAs and selected an optimal sensor sequence. As anticipated, the activation of reporter mRNA translation is HBV RNA sequence-specific and quantitatively correlates with the abundance of HBV RNA and cellular ADAR1 deaminase activity. The optimized HBV-RADARS system can be used with versatile reporter proteins in multiple HBV cell culture settings, including HBV replicon plasmid-transfected cells, cell lines expressing HBV RNAs from integrated transgenes, and cells infected with HBV. Particularly, using an antibiotic resistance gene as the reporter for HBV-RADARS allows for the phenotypic selection of HBV-infected HepG2 cells expressing human sodium taurocholate cotransporting polypeptide. Therefore, this HBV RNA-sensing reporter system is compatible with authentic HBV infection and can serve as a versatile platform for high-throughput screening of compounds that inhibit HBV infection and genome-wide genetic screens to identify cellular factors required for HBV infection of hepatocytes.

**IMPORTANCE:**

Traditional recombinant hepatitis B virus (HBV) reporter viruses are compromised in replication fitness and restricted to a single round of infection. HBV-RADARS, in principle, does not interfere with the viral life cycle as it acts through sensing the presence of HBV RNA and thus reports *de novo* HBV infection as well as the replication of transfected HBV replicons. The HBV-RADARS system represents a significant advancement in HBV research tool development and offers a replication-competent and highly adaptable reporter platform in live cells for genome-wide genetic and chemical screens. Hence, it opens new avenues for dissecting HBV-hepatocyte interactions and holds promises for the identification of host-encoded antiviral targets, thereby advancing efforts toward a functional cure for chronic HBV infections.

## INTRODUCTION

Even with an effective vaccine to prevent new infections, 254 million people worldwide still suffer from chronic hepatitis B virus (HBV) infections. HBV-infected patients are at high risk for developing end-stage liver disease, causing an estimated 1.1 million deaths per year (1, 2). Although the production of virus is suppressed by nucleos(t)ide analogs (NUCs), covalently closed circular (ccc) DNA, the template for the transcription of viral RNAs, persists in the nuclei of infected hepatocytes. Because of this limitation, a functional cure for the disease that yields sustained suppression of viremia and HBsAg loss and further mitigates the incidence of HBV-associated hepatocellular carcinoma is rarely achieved (3, 4). Hence, there is a pressing need to develop novel and curative anti-HBV therapy.

HBV enters hepatocytes by engaging its receptor, sodium taurocholate cotransporting polypeptide (NTCP) (5). Following viral entry, HBV nucleocapsid docks at the nuclear pore complex, and the viral relaxed circular DNA (rcDNA) genome is released into the nucleus upon nucleocapsid uncoating. Inside the nucleus, rcDNA can be recognized by cellular DNA repair machinery and converted into chromatinized cccDNA (6, 7). Transcription of cccDNA minichromosomes gives rise to four major RNA species, including the 3.5 kb pregenomic RNA (pgRNA) encoding HBV core protein (HBc) and viral polymerase (Pol), the 2.4 kb and 2.1 kb RNAs encoding HBV envelope proteins (HBs), and the 0.7 kb RNA encoding HBV X protein (HBx) with regulatory functions. Production of progeny virions begins with the packaging and reverse transcription of pregenomic RNA inside the nucleocapsid in the cytoplasm, followed by the synthesis of genomic rcDNA (6). Nucleocapsids enter the secretory pathway to acquire viral envelope proteins and eventually exit the infected hepatocytes as virions (8). Of particular significance for HBV persistence is the so-called retrograde transport of a portion of mature nucleocapsids to the nucleus early in an infection, which leads to the amplification of the cccDNA pool (6, 9).

The development of reporter-expressing systems for HBV infection has met limited success (10) because HBV has a small viral genome (3.2 kb) and does not tolerate large insertions (11). Moreover, the genome is tightly packed with four overlapping open reading frames (ORF), two enhancers, and four promoters. Therefore, inserting a reporter gene into the HBV genome almost inevitably disrupts viral gene expression and genome replication. Over the years, several studies reported different designs of recombinant HBV reporter viruses, such as replacing part of *core* ORF with NanoLuc, inserting DsRed fluorescent gene into *pol* ORF, duplicating the overlapping region of *core* ORF and *pol* ORF to allow insertion of blasticidin resistance gene, or adding a small tag into *pol* ORF (12–15). A common concept used in producing these reporter viruses is to modify HBV *core* or the spacer region of the *pol* gene so that it harbors a reporter gene. However, the yield of recombinant reporter viruses is often limited by sub-optimal replication efficiency (11, 16, 17). Moreover, the diminished HBV replication following initial infection results in a “one-round trip” for these recombinant reporter viruses. This has constituted a major obstacle to the implementation of high-throughput chemical and genome-wide genetic screens targeting HBV.

To overcome this limitation, we exploited reprogrammable adenosine deaminase acting on RNA sensors (RADARS) as a tool for the development of a live cell reporter system, which reports the presence and abundance of HBV RNA without disrupting HBV genome or its viral life cycle. RADARS technology was recently invented to selectively translate payload proteins based on sensing specific RNA species in living cells (18–20). Mechanistically, this approach harnesses the cellular double-stranded RNA (dsRNA) specific, A-to-I RNA editing enzyme adenosine deaminase acting on RNA (ADAR), to edit a UAG amber stop codon in a reporter messenger RNA into a UIG tryptophan codon. Protein translation is therefore made contingent on the hybridization of a specific target RNA to a corresponding sensor sequence, which then triggers ADAR-mediated A-to-I editing. Taking advantage of this system, we developed an HBV-RADARS reporter, which expresses reporter RNA with a UAG stop codon that is translated after sensing/base pairing with HBV RNA sequences. The HBV-RADARS reporter system bypasses the need to modify the viral genome and does not compromise viral fitness, allowing versatile reporter protein design.

## RESULTS

### Target site selection on the HBV genome and length optimization for RADARS

**Fig 1:**
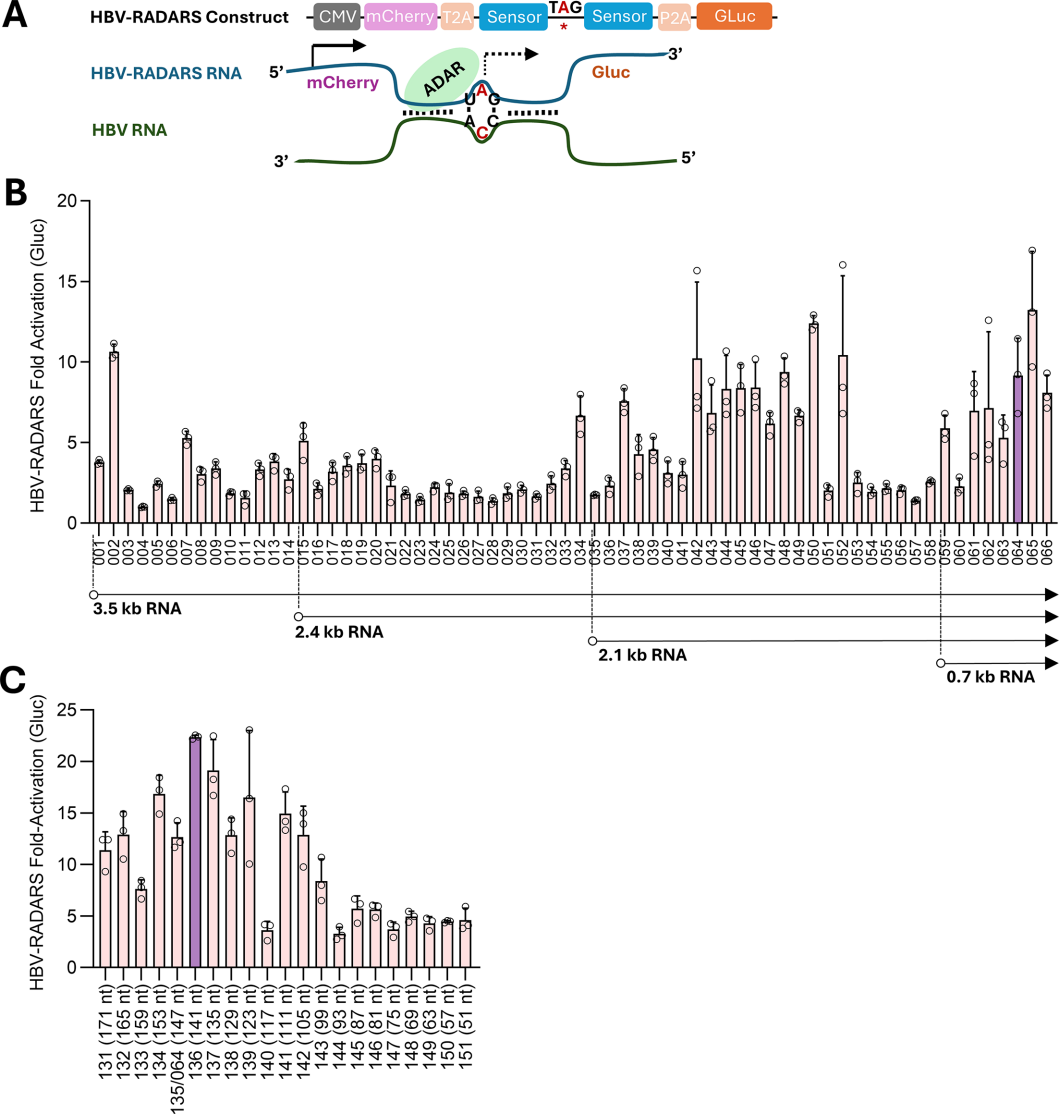
Screening of an optimal target sequence on HBV RNA for RADARS reporter. (**A**) Illustration of the HBV-RADARS reporter design. Expression of the Gluc reporter is blocked by the stop codon but gets rescued once the target RNA (HBV) hybridizes with the sensor RNA and triggers the ADAR-mediated A-to-I editing, resulting in the conversion of the stop codon into a readthrough codon. (**B**) HepG2-NTCP-C4 cells were transfected with HBV-RADARS plasmids that differ in the sensor sequences. Cells were either co-transfected with the pHBV1.3 plasmid expressing all HBV RNAs or the pCMV-GFP plasmid as a negative control. Five days after transfection, Gluc luminescence was measured, and the fold activation of HBV-RADARS was calculated as the relative ratio of Gluc levels in the presence of HBV plasmid versus control plasmid. The location of the CCA sites targeted by each HBV-RADARS was depicted by aligning the position of HBV RNA transcripts. Plasmid 064 targeting CCA1643 (highlighted in purple) was selected for further optimization. (**C**) HepG2-NTCP-C4 cells were transfected with the HBV-RADARS plasmids (plasmid 131 through 151) targeting CCA1643, varying the length of their sensor sequences (51–171 nts). Cells were either co-transfected with the pHBV1.3 plasmid or the pCMV-GFP plasmid as a negative control. Five days after transfection, Gluc luminescence was measured, and the fold activation of HBV-RADARS was calculated as the relative ratio of Gluc levels in the presence of HBV plasmid versus control plasmid. The HBV-RADARS plasmid 136 targeting CCA1643 with a 141-nt sensor was selected as prototype HBV-RADARS and is highlighted in purple. Mean ± SD is shown with three biological replicates.

To test the efficiency of the RADARS reporter system upon sensing HBV RNA, we initially designed a plasmid construct that contains an upstream mCherry ORF and a downstream Gaussia luciferase (Gluc) reporter ORF separated by ribosomal skipping sequences (T2A and P2A) and a 147-nt anti-HBV sensor sequence flanking a TAG stop codon (Fig. 1A). This construct allows constitutive mCherry expression, while it conditionally expresses a downstream Gluc reporter. Gluc expression requires that HBV RNA hybridizes to the sensor sequence triggering ADAR-dependent RNA A-to-I editing, converting the TAG stop codon into a TIG, which results in translation readthrough (Fig. 1A). Since an A:C mismatch flanked by long dsRNAs is the preferred ADAR target (21), we searched for the presence of 5′-CCA-3′ in HBV RNAs to design the 5′-TAG-3′ RADARS target and found 66 sites on HBV genotype D (GenBank: MF967563.1). Since RNA secondary structure, protein binding, and other sequence properties can influence ADAR editing efficiency (22), we systematically tested all ADAR targetable sites using co-transfection with a plasmid encoding HBV RNAs (pHBV1.3) or green fluorescent protein (GFP) RNA (pCMV-GFP) as a negative control, and calculated the Gluc fold activation to rank these target sites. The most efficient target sites were mapped to regions overlapping three of the four major HBV RNAs and to the 3′ end of all four major RNAs (Fig. 1B). The overall profile agrees with a study suggesting the RADARS sensor has a higher dynamic range when targeting the coding sequences of secreted proteins or the 3′ UTRs of transcripts (23). Individual validation of the most efficient constructs led to the selection of 1570–1716 (C1643) as the ideal target site, which targets an overlapping region of HBV RNA within the HBx ORF. Because the length of the RNA sensor has been shown to impact the downstream reporter activation likely through altering the hybridization strength with target RNA and ADAR recruitment (18, 21, 22), we further varied the anti-HBV sensor RNA lengths from 51 nts to 171 nts at 6-nt intervals and found that the HBV-RADARS construct harboring a 141-nt anti-HBV sensor sequence complementary to HBV 1573–1713 yielded the highest and most consistent Gluc activation, 22.4-fold (Fig. 1C). We therefore selected a prototype HBV-RADARS that senses the 1573–1713 (C1643) region of HBV RNA for further study.

**Fig 2:**
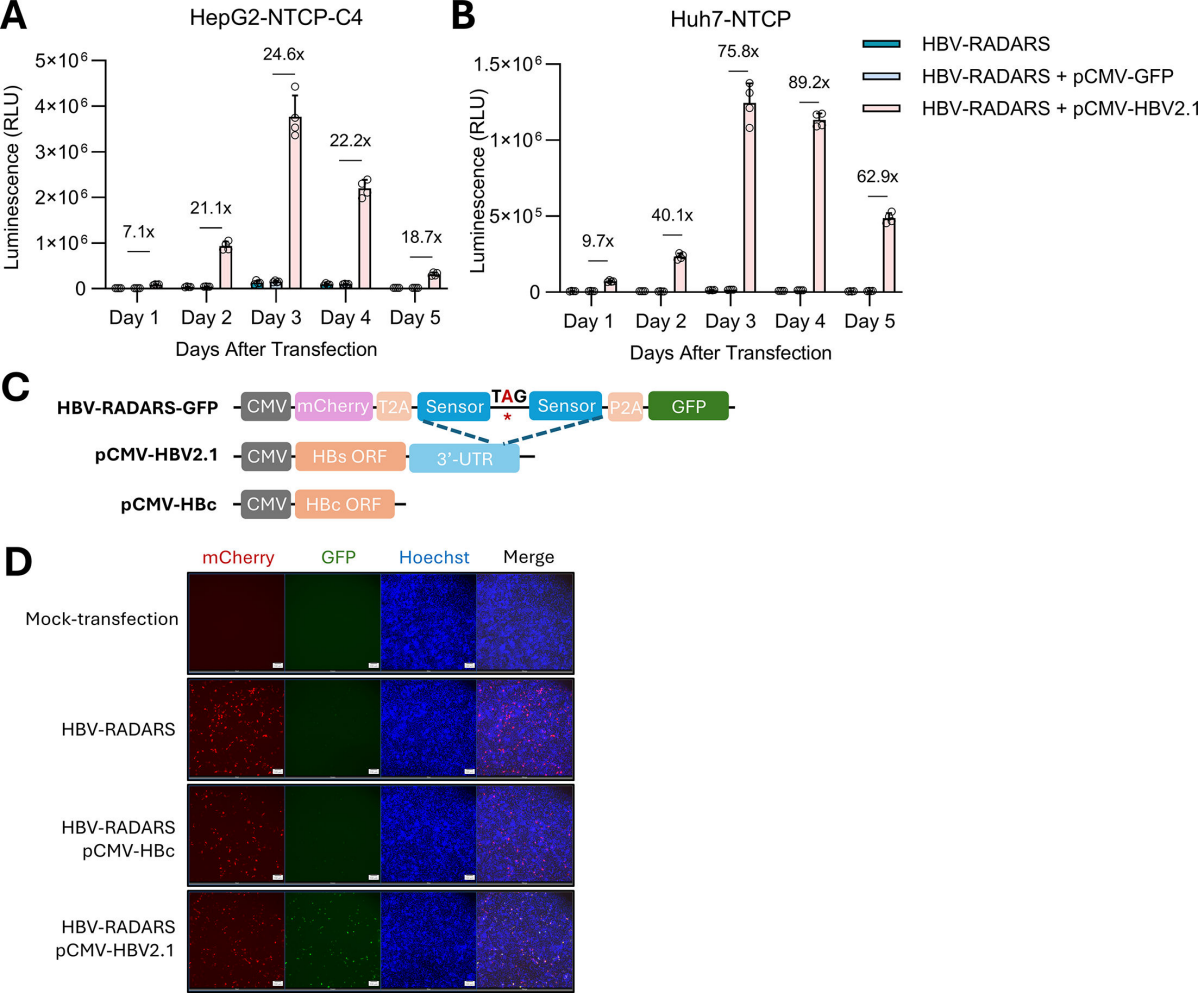
Validation of the prototype HBV-RADARS reporter. (**A and B**) HepG2-NTCP-C4 or Huh7-NTCP cells were either transfected with HBV-RADARS reporter plasmid, or with the negative control pCMV-GFP plasmid, or with the pCMV-HBV2.1 plasmid expressing the HBV 2.1 kb RNA containing the target region of the reporter plasmid. Gluc luminescence was measured from day 1 to day 5 after transfection. Fold activation of HBV-RADARS was calculated as the relative ratio of Gluc levels in the presence of pCMV-HBV2.1 compared with pCMV-GFP plasmid. Mean ± SD is shown with four biological replicates. (**C**) Physical maps of HBV-RADARS-GFP reporter with its target region on pCMV-HBV2.1, which is absent on pCMV-HBc. (**D**) HepG2-NTCP-C4 cells were mock-transfected or transfected with HBV-RADARS-GFP reporter plasmid alone, or together with the negative control pCMV-HBc plasmid, or with the pCMV-HBV2.1 plasmid expressing the HBV 2.1 kb RNA containing the target region. Four days after the transfection, cell nuclei were stained with 1 µg/mL Hoechst 33342 for 2 hours before imaging to monitor mCherry, GFP, and nuclei signals.

Transfection of the replication-competent HBV plasmid (pHBV1.3) produces HBx that is known to elevate episomal DNA transcription by degrading the SMC5/6 complex (24–26). To further verify the contribution of RNA editing to the activation of HBV-RADARS, a plasmid that expresses only the HBV 2.1 kb RNA (pCMV-HBV2.1) encoding HBV small envelope protein (HBs) was co-transfected with the HBV-RADARS plasmid. It resulted in peak activation of 24.6-fold and 75.8-fold at 3 days post-transfection in HepG2-NTCP-C4 and Huh7-NTCP cells, respectively (Fig. 2A and B). Furthermore, replacing the downstream Gluc reporter with a GFP reporter gives rise to green cells when co-transfected with pCMV-HBV2.1, whereas co-transfection with pCMV-HBc that lacks its target RNA region showed only basal GFP expression (Fig. 2C and D). Thus, efficient activation of the reporter can occur following expression of an HBV target transcript independent 0f HBx expression.

### Activation of HBV-RADARS is target RNA specific and correlates with the A-to-I RNA editing ratio

**Fig 3:**
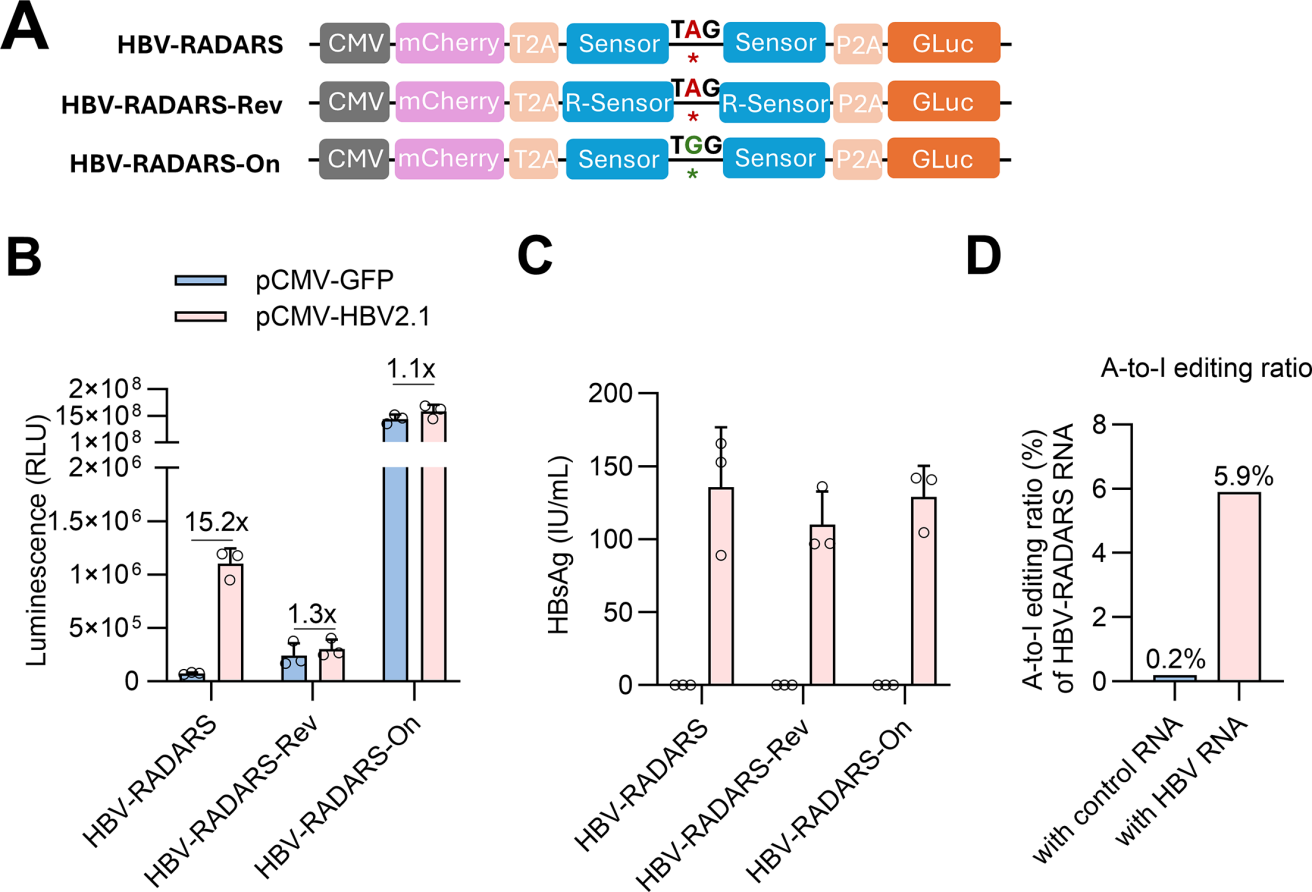
Activation of HBV-RADARS is target RNA sequence dependent. (**A**) Illustration of HBV-RADARS and control plasmids with the sensor regions highlighted. (**B**) HepG2-NTCP-C4 cells were transfected with the indicated plasmids and co-transfected with pCMV-HBV2.1 or pCMV-GFP. On day 4 after transfection, the Gluc luminescence was measured, and the fold activation of HBV-RADARS was calculated as the relative ratio of Gluc levels in the presence of pCMV-HBV2.1 versus pCMV-GFP plasmid. (**C**) The HBsAg secreted into culture supernatant from day 1 to day 4 after transfection was quantified with a chemiluminescent immunoassay. Mean ± SD is shown with three biological replicates. (**D**) The percentage of UAG converted into UIG within the HBV-RADARS RNA sensor region was quantified at 4 days post-transfection of HepG2-NTCP-C4, *via* RNA extraction, DNaseI digestion, reverse transcription, followed by next-generation sequencing-amplicon seq.

**Fig 4:**
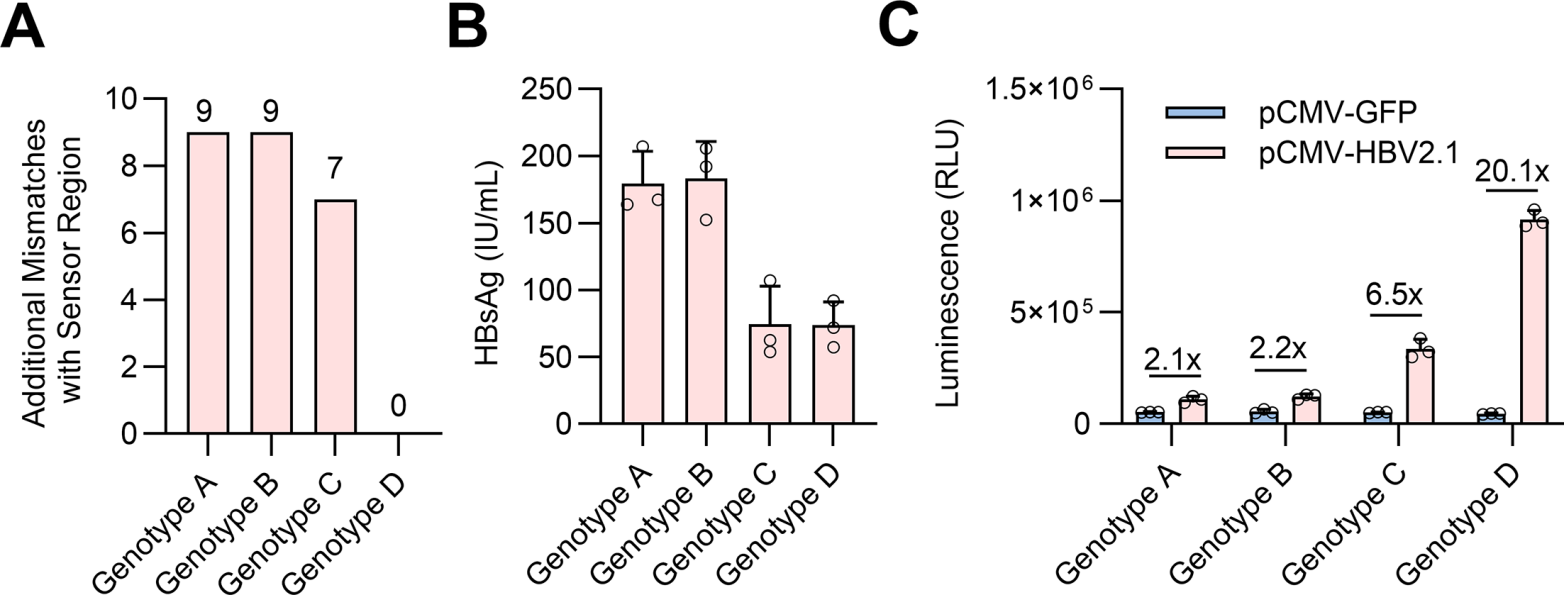
Characterization of HBV-RADARS in sensing HBV RNA derived from different genotypes. (**A**) Number of additional mismatches between the 141-nt HBV-RADARS sensor and the target regions from genotypes A, B, C, and D. (**B**) HepG2-NTCP-C4 cells were transfected with the HBV-RADARS plasmid and co-transfected with pCMV-HBV2.1 expressing HBV 2.1 kb RNA derived from HBV genotypes A, B, C, or D. Cells co-transfected with pCMV-GFP served as a negative control. The HBsAg secreted into culture supernatant from day 1 to day 4 after transfection was quantified with a chemiluminescent immunoassay. (**C**) Gluc luminescence was measured, and the fold activation of HBV-RADARS was calculated as the relative ratio of Gluc levels in the presence of pCMV-HBV2.1 versus pCMV-GFP plasmid. Mean ± SD is shown with three biological replicates.

To further verify the specificity controlling HBV-RADARS activation, we constructed a negative control plasmid designated as HBV-RADARS-Rev in which the HBV RNA sensor sequence was reversed and thus does not recognize HBV 2.1 kb RNA (Fig. 3A). Another control, the HBV-RADARS-On construct, has the TAG stop codon replaced with TGG so that the translation of the downstream reporter is constitutively active and does not depend on HBV RNA sensing and ADAR editing (Fig. 3A). As expected, only the HBV-RADARS construct, but not the other two control constructs, exhibited significant Gluc activation (15.2-fold) in response to comparable levels of HBV plasmid transfection in HepG2-NTCP-C4 cells (Fig. 3B and C). The ADAR deamination-dependent mechanism is further supported by a 30-fold increase in the A-to-I editing of HBV-RADARS RNA transcripts after co-transfection with pCMV-HBV2.1 plasmid encoding HBV RNA (Fig. 3D). To investigate the efficiency of the prototype HBV-RADARS construct in sensing different HBV genotypes, we generated additional HBV2.1 kb RNA expressing plasmids derived from HBV genotype A, B, and C (GenBank MN645903.1, MN645904.1, and MN645905.1). Interestingly, despite lower levels of HBsAg produced by pCMV-HBV2.1-Genotype D compared with genotypes A, B, and C, it nevertheless led to the highest Gluc induction, presumably due to the best sequence complementarity to the HBV-RADARS (Fig. 4). In contrast, HBV genotype A, B, and C yielded relatively less Gluc induction likely due to various degrees of additional mismatches, especially near the center TAG site that may cause misalignment of the dsRNA structure and thus impair the subsequent ADAR editing (Fig. 4 and Fig. S1). Taken together, these results demonstrated that HBV-RADARS function depends on specific HBV-derived target RNA sensing.

### Activation of HBV-RADARS is ADAR deaminase activity dependent

**Fig 5:**
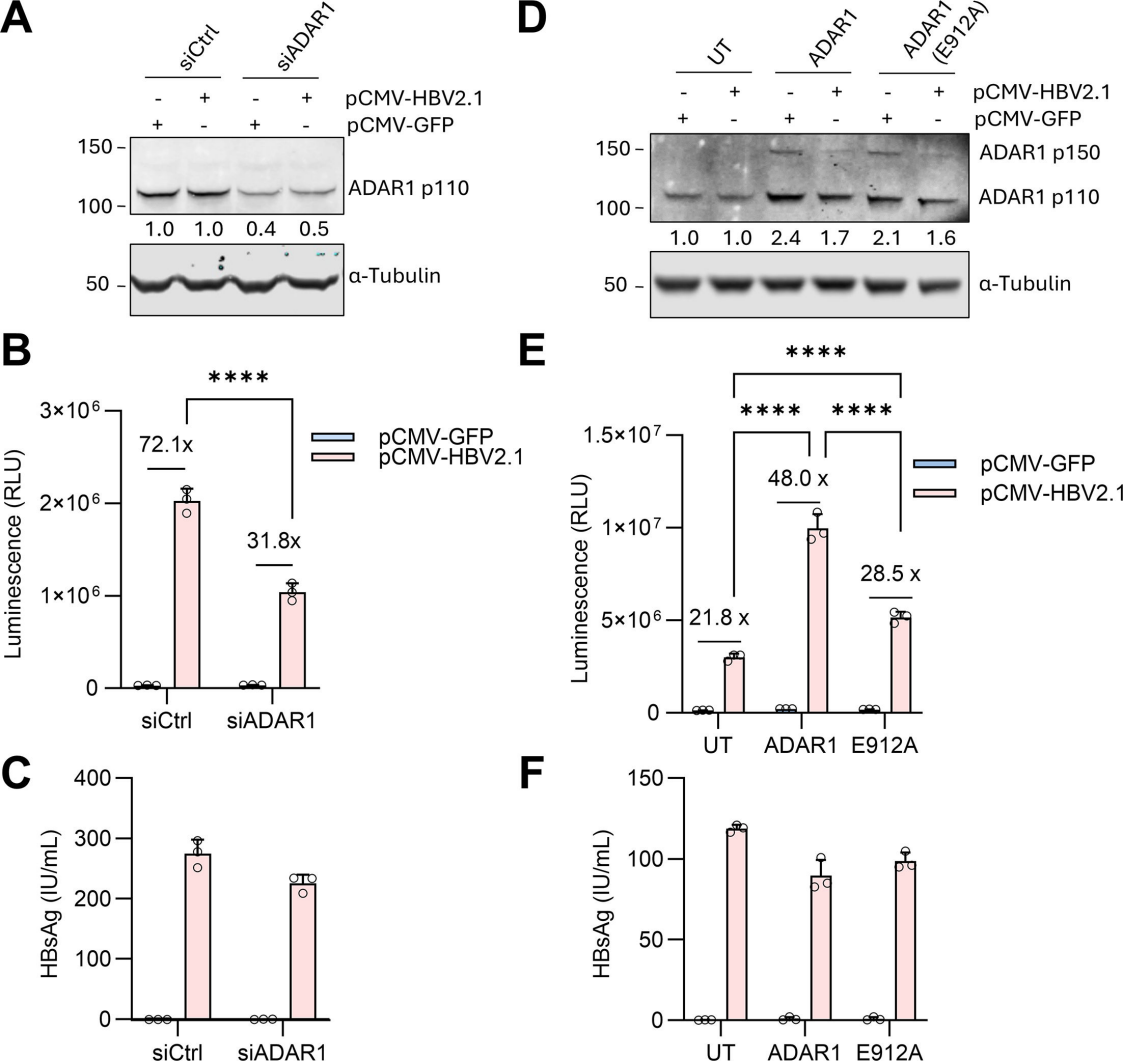
ADAR1 contributes to the activation of HBV-RADARS. (**A**) HepG2-NTCP-C4 cells were transfected with 10 nM scrambled siRNA (siCtrl) or siRNA against ADAR1 (siADAR1) by using Lipofectamine RNAiMAX. Simultaneously, cells were co-transfected with HBV-RADARS reporter plasmid and pCMV-HBV2.1 or with pCMV-GFP. Four days after siRNA and plasmid transfection, the protein levels of ADAR1 and α-tubulin were determined by Western blot analysis. Band intensities of ADAR1 p110 were analyzed and compared using Image Studio. (**B**) Gluc luminescence was measured, and the fold activation of HBV-RADARS was calculated as the relative ratio of Gluc levels in the presence of pCMV-HBV2.1 versus pCMV-GFP plasmid. (**C**) The HBsAg secreted into culture supernatant from day 1 to day 4 after transfection was quantified with a chemiluminescent immunoassay. (**D**) HepG2-NTCP-C4 cells were transfected with HBV-RADARS reporter plasmid with pCMV-HBV2.1 or with pCMV-GFP. In addition, ADAR1 or enzymatically inactive ADAR1 (E912A) was ectopically expressed in cells as compared to mock transfection (UT). Four days after plasmid transfection, the protein levels of ADAR1 and α-Tubulin were determined by Western blot analysis. Band intensities of ADAR1 p110 were analyzed and compared using Image Studio. (**E**) Gluc luminescence was measured, and the fold activation of HBV-RADARS was calculated as the relative ratio of Gluc levels in the presence of pCMV-HBV2.1 versus pCMV-GFP plasmid. (**F**) The HBsAg secreted into culture supernatant from day 1 to day 4 after transfection was quantified with the chemiluminescent immunoassay. Mean ± SD is shown with three biological replicates. ****, *P* < 0.0001.

ADAR1 is ubiquitously expressed across human tissues and cell types, and it has been implicated in RNA editing of hepatitis D virus in hepatocytes (27–29). Therefore, we next assessed the contribution of ADAR1 in the translational readthrough of the HBV-RADARS RNA. Notably, HepG2-NTCP-C4 cells showed detectable levels of the ADAR1 p110 isoform, which is constitutively expressed and localized in the nucleus (Fig. 5A), but not the interferon-inducible N-terminal extended ADAR1 p150 isoform (compare to Fig. 5D), which primarily localizes in the cytoplasm (30). Co-transfection of siRNA targeting ADAR1, which modestly reduced the amount of ADAR1 protein, proportionally attenuated the HBV RNA-triggered Gluc activation of HBV-RADARS (Fig. 5A through C). Conversely, the activation of HBV-RADARS is further enhanced when ADAR1 was overexpressed ectopically, which increases both ADAR1 p110 and ADAR1 p150 levels (Fig. 5D through F). However, the expression of ADAR1 mutant (ADAR1-E912A), which can bind dsRNA but lacks deaminase activity, resulted in significantly reduced enhancement (Fig. 5D through F) (31). Collectively, these data demonstrate that the prototype HBV-RADARS functions in an HBV RNA-sensing and ADAR1 RNA-editing-dependent manner.

### Activity of the HBV-RADARS correlates with HBV RNA expression levels

**Fig 6:**
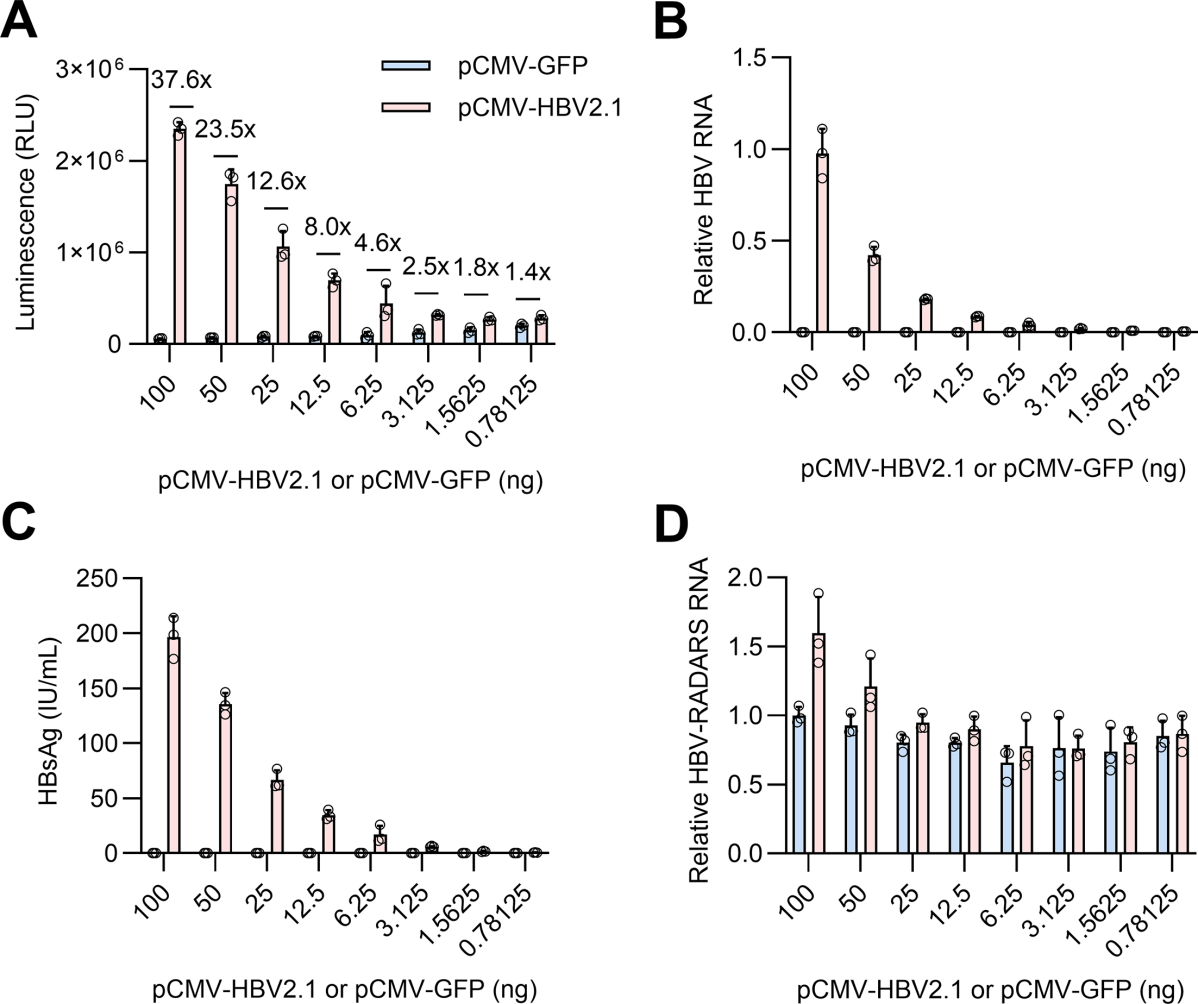
The level of HBV-RADARS activation correlates with the amount of target RNA. HepG2-NTCP-C4 cells seeded in a 48-well plate were transfected with 100 ng of HBV-RADARS plasmid and co-transfected with the indicated amounts of pCMV-HBV2.1 or pCMV-GFP for 4 days. (**A**) Gluc luminescence was measured, and the fold activation of HBV-RADARS was calculated as the relative ratio of Gluc levels in the presence of pCMV-HBV2.1 versus quantity-matched pCMV-GFP plasmid. (**B**) HBV 2.1 kb RNA transcripts were quantified by RT-qPCR and normalized to cellular β-actin RNA levels. Data are presented as HBV RNA levels relative to the 100 ng pCMV-HBV2.1 transfected group. (**C**) The HBsAg secreted into culture supernatant from day 1 to day 4 after each transfection condition was quantified with a chemiluminescent immunoassay. (**D**) HBV-RADARS reporter RNA transcripts were quantified by RT-qPCR using primers that target the mCherry region and normalized to cellular β-actin RNA levels. Data are presented as HBV-RADARS reporter RNA levels relative to the 100 ng pCMV-GFP transfected group. Mean ± SD is shown with three biological replicates.

To determine whether Gluc expression from HBV-RADARS correlates with the level of its target HBV RNA, we next co-transfected cells with various amounts of plasmid expressing HBV 2.1 kb RNA (pCMV-HBV2.1), matched with the same amounts of GFP plasmid (pCMV-GFP) as controls. As expected, increasing the amount of pCMV-HBV2.1 plasmid during co-transfection generated more HBV 2.1 kb RNA in a dose-dependent fashion (Fig. 6B) and thereby higher levels of HBsAg (Fig. 6C), which led to an increasing Gluc activation from comparable levels of HBV-RADARS mRNA (Fig. 6A through D). The correlation between HBV-RADARS activation and its target HBV RNA abundance was further tested in HepG2.2.15 cells that harbor a genotype D HBV transgene and thus constitutively express all major HBV RNA species (32). Treating cells with the HBV RNA destabilizing compound RG7834 (except for HBx mRNA) (33–35) significantly reduced total HBV RNA and antigen expression, corresponding with a decrease in HBV RNA-mediated Gluc activation from HBV-RADARS reporter (Fig. S2).

Endogenous dsRNA and virus-derived dsRNA can potentially activate cellular dsRNA sensors, leading to ISG expression, such as IFN-β (36). Since the mechanism of HBV-RADARS reporter involves dsRNA formation, which is a potential trigger for innate immune activation, we tested IFN-β RNA levels in HBV-RADARS and pCMV-HBV2.1 co-transfected cells. The lack of significant IFN-β induction suggests that at least in these cells, the HBV-RADARS activation is not accompanied by dsRNA-mediated innate immune responses (Fig. S3A). This result aligns with the original RADARS study, which showed no activation of the dsRNA-sensing pathway across a panel of human cell lines, including HepG2 (18).

### HBV-RADARS responds to authentic HBV infection of HepG2-NTCP

**Fig 7:**
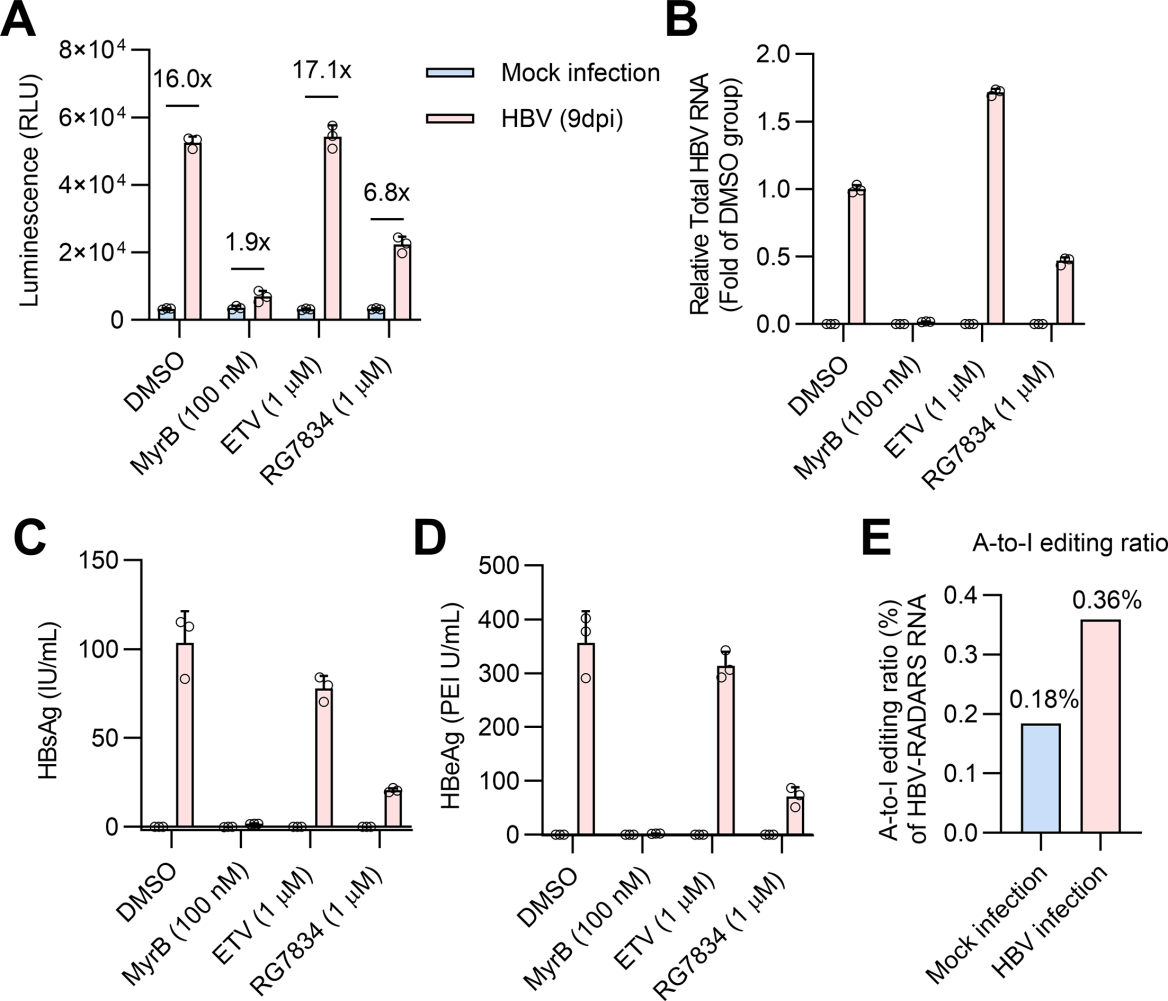
HBV-RADARS reporter is effective in *de novo* HBV-infected cells. HepG2-NTCP-C4 cells were transfected with HBV-RADARS 1 day prior to HBV infection, followed by mock-infection or infection with HBV at 5,000 genome equivalents per cell (5,000 GEs/cell). MyrB was added during viral inoculation. One day after viral inoculation, cells were either left untreated (DMSO) or treated with ETV or RG7834 until 9 dpi. Medium was refreshed every other day. (**A**) Gluc luminescence was measured, and the fold activation of HBV-RADARS was calculated as the relative ratio of Gluc levels in the presence of HBV versus mock infection. (**B**) Total HBV RNA transcripts were quantified by RT-qPCR and normalized to cellular β-actin RNA. Data are presented as HBV RNA levels relative to the infected and untreated (DMSO) group. (**C and D**) HBsAg and HBeAg secreted into culture supernatant from 7 dpi to 9 dpi were quantified with the chemiluminescent immunoassay. Mean ± SD is shown with three biological replicates. (**E**) The percentage of UAG converted into UIG within the HBV-RADARS RNA sensor region was quantified at 9 dpi, *via* RNA extraction, DNaseI digestion, reverse transcription, followed by NGS-amplicon seq.

To determine whether HBV-RADARS can also sense HBV RNA derived from infection of permissive cells with HBV, we first transfected HepG2-NTCP-C4 with the HBV-RADARS plasmid, followed by infection with 5,000 genome equivalents (GE) of HBV per cell. Nine days post-infection, luciferase activity was determined. HBV infection-mediated Gluc expression from HBV-RADARS was approximately 16-fold greater than that in mock-infected cells (Fig. 7A). Blocking the HBV receptor with the entry inhibitor MyrB efficiently blocked the reporter luciferase activation, whereas reduction of HBV RNA (except for HBx mRNA) by the HBV RNA destabilizing compound RG7834 partially ablated the reporter activation. As expected, the HBV RNA reverse transcription inhibitor ETV did not alter the reporter activation because it did not reduce HBV RNA (Fig. 7A through D). Importantly, HBV infection led to a twofold (0.36% over 0.18%) increase in the A-to-I editing of HBV-RADARS RNA transcripts (Fig. 7E). Consistent with the results from HBV plasmid-transfected cells, no significant IFN-β induction was detected with HBV-RADARS activation in HBV-infected cells (Fig. S3B). In agreement with HBV-RADARS luciferase reporter, the HBV-RADARS-GFP reporter also exhibited elevated GFP expression specific to HBV infection versus mock (Fig. S4). Notably, the overall GFP induction and the increase in A-to-I editing ratio by HBV infection of HepG2-NTCP-C4 cells are not as strong as expected (see Discussion); we therefore attempted overexpression of ADAR1 protein. However, while excessive amounts of ADAR1 increased the GFP-positive cell ratio, it also led to significant non-specific expression of GFP in mock-infected cells, likely due to promiscuous ADAR1 off-target editing events as reported by other ADAR1 overexpression-based approaches too (Fig. S4) (18, 37). Taken together, the results demonstrated the expected relationship between HBV RNA expression levels and reporter activity in the HepG2-NTCP HBV infection system.

### HBV-RADARS expressing an antibiotic-resistant gene allows for positive selection of HBV-infected HepG2-NTCP cells

**Fig 8:**
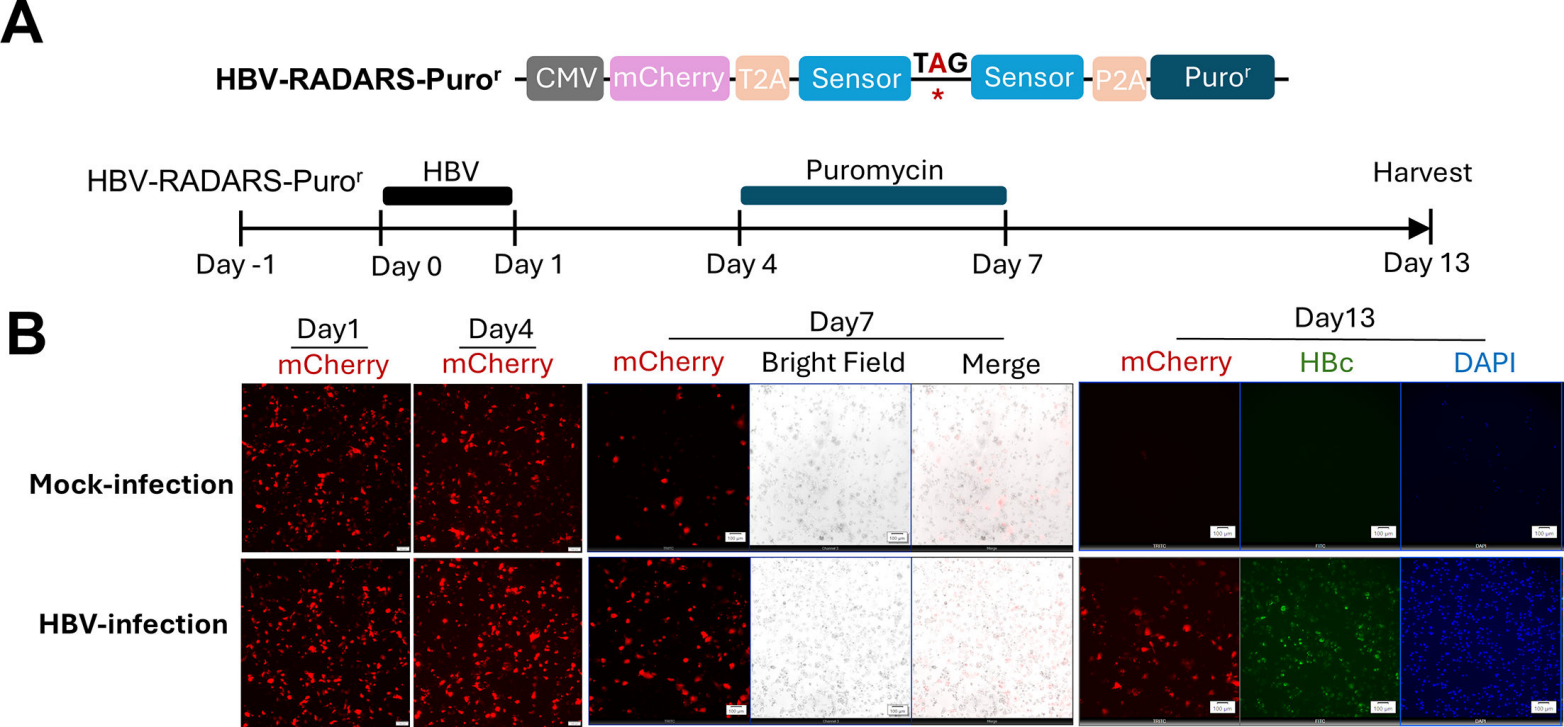
HBV-RADARS reporter enables phenotypic selection of HBV-infected cells. (**A**) HepG2-NTCP-C4 cells were transfected with HBV-RADARS reporter plasmid conditionally expressing the puromycin resistance gene. One day after plasmid transfection, cells were mock-infected or infected with HBV at 5,000 GEs/cell. Cells were exposed to 2.5 µg/mL puromycin from 4 dpi to 7 dpi and then cultured in puromycin-free medium until 13 dpi before harvesting and staining for HBc. (**B**) Fluorescent and bright field images were taken at the indicated timepoints. On the day of harvesting, cells were subjected to HBc immunofluorescence assay. Images in each condition are representative of three independent experiments.

Next, we tested whether an HBV-RADARS reporter conditionally expressing an antibiotic-resistant gene permits the selection of HBV-infected cells. This phenotypic assay could enable genetic screens to identify elusive host factors controlling HBV replication. Accordingly, we replaced the Gluc gene with a puromycin resistance gene and designated it as HBV-RADARS-Puro^r^ (Fig. 8A). Transfection of the plasmid into HepG2-NTCP-C4 cells gave rise to a comparable level of mCherry expression from the upstream of the reporter at 1 day post-HBV infection versus mock infection (Fig. 8B). Notably, the mCherry level was slightly higher in HBV-infected cells after 4 dpi, presumably due to HBx and its transactivation function to promote the HBV-RADARS-Puro^r^ plasmid expression (24, 25). After HBV infection establishment at 4 dpi, cells were transiently exposed to 2.5 μg/mL puromycin for 3 days and cultured to 13 dpi, which led to the elimination of the vast majority (>99%) of mock-infected cells (Fig. 8B). Importantly, however, a substantial number (about 5%–10%) of HBV-infected cells survived puromycin selection and stained positive for HBc, indicating that they had a reasonably high level of HBV RNA as well as HBV-RADARS-Puro^r^ to activate the puromycin-resistant gene during the selection phase (Fig. 8B). Therefore, these results demonstrated the feasibility of using this live cell HBV reporter system for phenotypic selection of HBV RNA-positive cells, promising to make it a versatile molecular tool for chemical and genetic screens, as well as selective targeting of HBV-infected cells.

## DISCUSSION

RADARS are based on the conditional protein translation activated by the sensing of specific RNA sequences in living cells (18–20). In this study, we adapted RADARS to HBV *in vitro* culture systems by developing an HBV-RADARS reporter through scanning different target regions on HBV RNA and optimizing the length of HBV RNA sensor (Fig. 1). Our results indicate that the HBV-RADARS reporter activation is target sequence specific (Fig. 3 and 4), and the level of reporter activation correlates with the amount of target RNA present in cells (Fig. 6 and 7, and Fig. S2). Comparing with conventional HBV reporter systems, which are usually based on replacing part of the HBV genome by a reporter gene and then supplementing the disrupted viral gene *in-trans* to generate recombinant viruses (13, 15), HBV-RADARS reporter has several key advantages. First, it can be used with wild-type HBV, supporting unperturbed viral replication and infectious progeny production. Second, although HBV has a small viral genome (3.2 kb) and does not tolerate large insertions, HBV-RADARS can be designed to express versatile reporter proteins such as Gluc, GFP, puromycin-resistant gene, etc*.* Third, while recombinant HBV reporter viruses are difficult to produce due to a strong *cis*-preference and genome size limitation during HBV replication (11, 16, 17), HBV-RADARS reporter is relatively straightforward to generate, as it can be expressed from a plasmid and, in principle, other expression platforms as well, thereby offering a versatile and adaptable tool for diverse applications.

Sensing of dsRNA by host pattern recognition receptors constitutes a major cell innate immune defense mechanism. Activation of dsRNA sensors can lead to global translational shutdown, IFN responses, and inflammatory cytokine responses (36, 38). Although HBV-RADARS reporters involve the formation of dsRNA between reporter RNA and HBV RNA, we did not detect discernible protein expression changes, such as HBsAg, Gluc (Fig. 3 and 5), and ADAR1 (Fig. 5), nor did we find significant induction of IFN-β RNA (Fig. S3). This is potentially due to the fact that ADAR-mediated RNA editing is known as a general suppressive mechanism to attenuate cellular dsRNA responses, in addition to the intrinsically weakened dsRNA responses in hepatoma cells (39–41). Further studies are needed to fully understand the dynamics of dsRNA formation and its immunostimulatory potential for a more comprehensive assessment.

As anticipated, the activation of HBV-RADARS reporter is ADAR deaminase dependent (Fig. 5). Therefore, it is conceivable that the amount and functionality of ADAR in target cells will ultimately impact the degree of HBV-RADARS activation. This probably contributes to the higher Gluc-activation observed in Huh7-NTCP over HepG2-NTCP-C4 cells (Fig. 2). Of note, the HBV-RADARS reporter is effective even with endogenous ADAR levels. This is important because while overexpression of ADAR1 enhances the reporter activation, it also leads to elevated non-specific reporter expression in the absence of target RNA (Fig. S4). Such an observation is consistent with the higher RADARS off-target rate upon ADAR overexpression indicated by whole-genome sequencing (18).

Interestingly, when HBV-RADARS is introduced as a plasmid reporter, it undergoes HBx-mediated episomal DNA transcriptional activation upon HBV infection (24, 25), which further aids in the HBV-specific reporter expression. The time-dependent, elevated mCherry expression (upstream of the stop codon) in HBV-infected cells can reflect the contribution of HBx (Fig. 8 and S4). However, the RNA editing mechanism still contributes significantly to reporter activation based on the increased A-to-I editing ratio after HBV infection (Fig. 7E) as well as the reduced Gluc activation following RG7834 treatment that reduces all major HBV RNA species but not HBx mRNA (Fig. 7) (35, 42).

Notably, it appears that our prototype HBV-RADARS can more efficiently detect HBV RNA produced by plasmid transient transfection than by HBV infection in HepG2-NTCP-C4 cells (Fig. 6 and 7). In fact, a very high amount of viral input (5,000 GEs/cell) is needed to achieve ideal reporter activation, which is a limitation of this approach. This is to best overcome the lower HBV RNA levels produced by only a few copies of cccDNA present per infected cell. Another possibility might be the sub-optimal transfection and infection rate. It is conceivable that a significant proportion of HBV-RADARS plasmid-transfected cells are not infected by HBV. Engineering of HepG2-NTCP-C4 cells that constitutively express HBV-RADARS RNA through lentiviral transduction was not successful either due to low basal reporter expression when compared to transient transfection (Fig. S5). Alternatively, our Western blot results indicate that the nuclear form of ADAR1 (ADAR1 p110) is the major isoform in HepG2-NTCP-C4 cells (Fig. 5); therefore, RNA editing that occurs co-transcriptionally in the nucleus could be the most efficient way to activate reporter expression in our cell cultures. Since co-transfection of reporter and HBV plasmids likely delivers high amounts of these two plasmid populations into the nuclei within close distance, it is intrinsically more efficient than HBV infection, where HBV RNA is derived from cccDNA, which could localize and transcribe at very specific nuclear regions (43, 44). The possible temporal and spatial factors that impact HBV-RADARS reporter in the HBV infection system warrant further investigation.

It should be acknowledged that the full utility of the HBV RADAR reporter system depends on increasing sensitivity and specificity in infection systems. The HBV sensor sequence in the current prototype HBV-RADARS design may theoretically be improved. This is based on the incomplete A-to-I editing of HBV-RADARS RNA (Fig. 3 and 7), and the HBV-RADARS-On readthrough construct had about 100-fold higher Gluc expression than the activated HBV-RADARS (Fig. 3). In the meantime, although minimal, both Gluc and GFP-expressing HBV-RADARS reporters exhibit leaky expression even in the absence of target RNA species (Fig. 2A and D), presumably due to non-specific ADAR editing or RNA splicing that overcomes the stop codon. Furthermore, another recent technical advancement in RADARS design, which utilizes the folding of tertiary RNA structures on the reporter RNA to bypass the need for the CCA site on the target RNA, results in broadened target site selection (21). Therefore, it is entirely possible that future designs leveraging better structural prediction of specific dsRNA between sensor and target RNA, as well as inserting additional stop codons as translational gatekeepers, may further improve the sensitivity and specificity of HBV-RADARS, which is essential when implementing HBV-RADARS as a therapeutic platform to selectively target HBV RNA-positive cells.

Finally, our proof-of-concept study demonstrated that the HBV-RADARS reporter expressing an antibiotic-resistant gene marker allows phenotypic selection of HBV-infected cells (Fig. 8). This selection will potentially enable unbiased genome-wide genetic screens and large-scale small molecule chemical screens, which have been challenging for HBV research in the past.

In summary, this live cell HBV-RADARS reporter has the potential to fill important gaps in knowledge concerning host-virus interactions critical for the HBV life cycle and ultimately provide new targets for antiviral therapies to achieve a functional cure for patients suffering from chronic hepatitis B infection.

## MATERIALS AND METHODS

### Cell culture

Human hepatoblastoma cell line HepG2-NTCP-C4 was kindly gifted from Dr. Koichi Watashi (45) at Japan National Institute of Infectious Diseases and cultured in DMEM/F12 (1:1) supplemented with 10% fetal bovine serum (FBS) (Gibco), 100 U/mL of penicillin, 100 µg/mL of streptomycin, and 500 µg/mL G418 (Gibco). HepG2.2.15 (32) was maintained the same way as HepG2-NTCP-C4. Huh7-NTCP was engineered by transducing Huh7 (gifted from Dr. Ju-Tao Guo, Blumberg Institute) with human NTCP expressing retroviruses (packaged from pCX4bsr-NTCP and pVSVG transfected GP2-293 cells) and selecting under 10 µg/mL Blasticidin (Gibco) for 2 weeks. Huh7-NTCP was cultured in DMEM medium supplemented with 10% FBS (Gibco), 100 U/mL of penicillin, 100 µg/mL of streptomycin, and 5 µg/mL Blasticidin. HepAD38 cells were obtained from Dr. Christoph Seeger from Fox Chase Cancer Center, Philadelphia, USA (46), and cultured in DMEM/F12 (1:1) supplemented with 10% FBS (Gibco), 100 U/mL of penicillin, 100 µg/mL of streptomycin, and 1 µg/mL tetracycline (Sigma). Tetracycline was removed from HepAD38 culture media to initiate pgRNA transcription and HBV production as needed. All cells were maintained in 5% CO_2_ incubators at 37˚C. All cell culture experiments were performed in 50 µg/mL rat tail collagen (Corning) coated plates.

### Plasmids

The HBV-RADARS plasmid was generated as follows: First, an intermediate plasmid containing mCherry-P2A-Sensor-T2A-Gluc was generated by digesting the backbone plasmid pDY1243-Fluorescent RADARS (Addgene #182540) with BsrGI and NotI, followed by assembly with a synthesized DNA fragment containing P2A-T2A-Gluc sequences (designated as F RD000K, IDT, Table S2) using the NEBuilder HiFi DNA Assembly Master Mix (New England Biolabs). Next, the RADARS expression cassette was PCR amplified with primers F1 and R1 (Table S1), and cloned into the pENTR4 backbone derived from a pEN-H133A plasmid (47) digested with PspXI and HindIII, to yield the second intermediate plasmid pEN-RADARS. Finally, a series of HBV RADARS plasmids (HBV-RADARS-001 to -066) containing various HBV sensor sequences targeting different CCA sites present in HBV genotype D RNA (GenBank: MF967563.1) were constructed by amplifying pEN-RADARS with primers F2 and R2 (Table S1) to obtain the linear plasmid backbone, which was assembled with different 147-nt synthesized sensor fragments (Table S2, IDT eBlock). Similarly, HBV-RADARS-131 to -151 were designed to screen for the optimal sensor length, using the pEN-RADARS backbone, PCR amplified with F3 and R3, and assembled with various sensor sequences (Table S2, IDT eBlock).

Plasmids HBV-RADARS-Rev with reversed sensor sequence to the prototype HBV-RADARS, and plasmid HBV-RADARS-On containing TGG readthrough sensor sequence were derived from backbone PCR of HBV-RADARS with primers F1 and R1 (Table S1), and fusion with synthetic sensor sequences (Rev Sensor and On sensor, IDT), respectively, using NEBuilder HiFi DNA Assembly. Plasmids HBV-RADARS-GFP and HBV-RADARS-Puro^r^ have the Gluc sequence replaced with the eGFP sequence and puromycin-resistant gene sequence, respectively. Plasmids were constructed by PCR amplification of the HBV-RADARS backbone with primers F4 and R4, insertion of eGFP synthesized from IDT (Table S2), or insertion of Puro^r^ that is PCR amplified from pLVX-Puro (Takara #632164) using primer F5 and R5 (Table S1).

Several HBV expression constructs were used in this study. The pHBV1.3 plasmid and pCMV-HBc plasmid are kind gifts from Ju-Tao Guo (Blumberg Institute) and were previously described (48). pCMV-HBV2.1 (genotype D) was a kind gift from Tianlun Zhou (Blumberg Institute). To generate plasmids expressing HBV genotype A, B, and C RNA transcripts, a set of intermediate plasmids containing full-length HBV was constructed by PCR amplifying pTRE-HBV plasmid backbone (49) with primers F6 and R6 (Table S1), and assembling the linear backbone with synthesized fragments A1/A2, B1/B2, or C1/C2 (Table S2, IDT). Then, the pCMV-HBV2.1-GA, -GB, and -GC plasmids were generated by PCR amplifying the pCMV-HBV2.1-GD backbone with primers F7 and R7 (Table S1), and assembling with the HBV 2.1 kb regions derived from pTRE-HBV-GA,-GB, and GC (PCR by primers F8 and R8, F9 and R9, and F10 and R10, respectively).

To express ADAR1 and its mutant form, HBV-RADARS backbone (pEN) was PCR amplified with F11 and R11 to introduce an HA-tag. The coding sequences for ADAR1 p150 were PCR cloned from pmGFP-ADAR1-p150 (Addgene #117927) using primers F12 and R12, and ADAR1 p150 E912A mutant was cloned using the same backbone to assemble with two overlapping fragments, which are generated by pmGFP-ADAR-p150 plasmid PCR with F12 and R13, and with F13 and R12 (Table S1), to introduce the E912A mutation. The resulting plasmids are designated as pEN-HA-ADAR1 and pEN-HA-ADAR1-E912A.

All plasmid constructs were assembled using the NEBuilder HiFi DNA Assembly Master Mix. Primers used for construction of these plasmids are listed in Table S1. All self-constructed plasmids were confirmed by sequencing.

### Antibodies, chemicals, and siRNAs

Anti-ADAR1 antibody and anti-α-Tubulin antibody were purchased from Cell Signaling Technology. All antibodies were used at 1:1,000 dilutions for Western blot assays. Anti-HBc antibody (C1-5) was purchased from Santa Cruz Biotechnology and used at 1:100 dilution for the immunofluorescence assay. MyrB, ETV, and RG7834 were all purchased from MedChemExpress. Puromycin and Blasticidin were purchased from Gibco. Hoechst 33342 was purchased from Thermo Fisher. DAPI was purchased from Cell Signaling Technology. ADAR1 siRNA (ON-TARGETplus siRNA SMARTpool) and scrambled control siRNAs (ON-TARGETplus non-targeting control pool) used in this study were purchased from Dharmacon.

### Plasmid transfection and siRNA transfection

Plasmid transfections were carried out using Lipofectamine-3000 (Invitrogen) based on the manufacturer’s instructions. For experiments involving HBV plasmid transfection (unless otherwise indicated), 200 ng DNA of HBsAg-expressing plasmid and 100 ng DNA of HBV-RADARS plasmid were resuspended in 30 µL Opti-MEM reduced serum medium (Gibco) containing 0.9 µL P3000. The DNA mixture was then added to 30 µL Opti-MEM containing 0.9 µL Lipo-3000, mixed, and incubated at room temperature for 15 min. The DNA-liposome suspension was added to 70%–80% confluent cells cultured in 500 µL Opti-MEM medium in a 24-well plate. Transfection medium was removed 6 h after and refreshed with regular DMEM/F12 growth medium. For experiments involving HBV infection, 200 ng HBV-RADARS plasmid was used to transfect cells in 24-well plates. For experiments involving ectopic expression of ADAR1, 200 ng ADAR1 or control plasmid was used to transfect cells in a 24-well plate.

siRNA transfections were carried out using Lipofectamine RNAiMAX (Invitrogen) based on the manufacturer’s instructions. For one well of a 24-well plate, 5 pmol siRNA oligonucleotides and 1 µL Lipofectamine RNAiMAX were used to generate siRNA-liposome suspension in Opti-MEM medium at room temperature for 15 min, followed by transfecting cells seeded at 70%–80% confluency to have a final siRNA concentration at 10 nM. Transfection medium was removed 6 h after and refreshed with regular DMEM/F12 growth medium.

### Gaussia luciferase assay

Medium containing secreted luciferase was collected 96 h after HBV-RADARS plasmid transfection, unless otherwise noted. 10 µL of culture medium was used to measure luciferase activity using Gaussia Luciferase Assay Kits (Thermo Fisher #16161) on a Tecan plate reader following the manufacturer’s protocol. The absolute Gluc values were directly read out from the Tecan plate reader.

### HBsAg and HBeAg chemiluminescent immunoassays

The HBsAg and HBeAg that are secreted into the culture supernatant following HBV infection or plasmid transfection were collected, diluted threefold, and subsequently used for quantification using HBsAg and HBeAg CLIA kits (Ig Biotechnology) following the manufacturer’s protocol. For HepG2.2.15, HBsAg and HBeAg levels were measured directly without dilution. The final concentrations were calculated by correlating to the standard concentration curves and multiplying by the dilution factors.

### Western blot assay

Cells grown in a 24-well plate were lysed with 100 µL 1× NuPAGE LDS sample buffer supplemented with 2.5% 2-mercaptoethanol. Cell lysate was boiled on a heat block at 100°C for 20 min, briefly spun, and then resolved by running in NuPAGE 4-12% Bis-Tris Gel (Thermo Fisher) with MOPS-SDS Running Buffer. Proteins were transferred from the gel onto a PVDF membrane using the iBlot 2 Dry Blotting System. Membranes were blocked in Intercept Blocking Buffer (LI-COR) at room temperature for 1 h and then incubated with indicated antibodies in Intercept T20 Antibody Diluent (LI-COR) at 4°C overnight. After washing with TBST (TBS + 0.1% Tween 20), the membrane was incubated with LI-COR IRDye secondary antibodies in Intercept T20 Antibody Diluent (LI-COR) at room temperature for 1 h. Membranes were again washed with TBST and imaged with the LI-COR Odyssey system.

### RNA extraction and qPCR analysis

Total RNA was extracted with NucleoZOL (Takara) according to the manufacturer’s instructions. The potential plasmid DNA contaminants were digested by incubating samples with 2.5 μL DNaseI (New England Biolabs) at 37°C for 1 h, followed by heating at 75°C for 10 min to inactivate DNaseI. RNA sample quality was measured by UV absorbance at 260, 280, and 230 nm with a NanoDrop 2000 Spectrophotometer (Thermo Scientific). Detection of HBV-RADARS RNA, HBV RNA, and cellular RNAs was conducted using SuperScript III Platinum One-Step qRT-PCR kit (Invitrogen). Real-time PCR assays were performed using a LightCycler 480 II (Roche) with primers listed in Table S1. The qRT-PCR program was performed following the manufacturer’s protocol.

### A-to-I editing ratio analysis

After total RNA extraction, residual HBV-RADARS plasmid DNA contamination was digested by incubating with 5 µL DNaseI (New England Biolabs) at 37°C for 1 h, followed by heating at 75°C for 10 min to inactivate DNaseI. Then, the Sensor region of the HBV-RADARS RNA was PCR amplified using a cDNA One-step kit (NEB) with the primers F14 and R14 (Table S1), during which the adapter sequences for next-generation sequencing (NGS) amplicon seq were also ligated to the DNA fragment. The intended DNA amplicon was retrieved by 1.5% agarose gel electrophoresis based on the calculated size, purified by QIAquick Gel Extraction Kit (Qiagen), and submitted to NGS amplicon sequencing services to count reads that contain TAG or TGG (after ADAR editing) in the middle of the sensor region.

### HBV virion production, titer determination, and HBV infection assay

Genome type D HBV virion was generated from collecting the culture media (DMEM containing 3% FBS, 1× NEAA, and 100 U/mL of penicillin, 100 µg/mL of streptomycin) of HepAD38 cells for about 8 weeks after removing tetracycline and subsequently concentrated for 200-fold (volume) by precipitating with 8% PEG-8000 (Sigma) at 2,000 × *g* for 15 min and resuspending in Opti-MEM medium. Genome equivalents were determined by subjecting a small aliquot of virus stock to DNA extraction using the HBV core DNA extraction method followed by HBV DNA qPCR quantification (50). HBV plasmid pHBV1.3 with serial dilutions was used for generating the standard curve and calculating the genome equivalents. For HBV infection, HepG2-NTCP-C4 cells that were seeded confluently in collagen-coated plates were pretreated with DMEM medium supplemented with 3% FBS, 1× NEAA, 2% DMSO, and 100 U/mL of penicillin, 100 µg/mL of streptomycin for 24 h. Cells were then infected with HBV at 5,000 genome equivalents per cell in pretreatment media added with 4% PEG-8000 (Sigma). The inoculums were removed at 24 h post-infection and cells were washed with PBS 3–5 times and kept in DMEM medium supplemented with 3% FBS, 1× NEAA, 2% DMSO, and 100 U/mL of penicillin, 100 µg/mL of streptomycin.

### Immunofluorescence assay

HepG2-NTCP-C4 cells were fixed with PBS pH 7.4 containing 4% paraformaldehyde (Thermo Fisher), followed by 10 min incubation with 0.25% Triton X-100. Cells were then blocked by a 30 min incubation at room temperature with 1% BSA, 22.52 mg/mL glycine in PBST (PBS + 0.1% Tween 20). Next, cells were incubated with 1:100 diluted HBc antibody in 1% BSA in PBST (PBS + 0.1% Tween 20) overnight at 4°C. Bound primary antibody was visualized by using Alexa Fluor 488-conjugated secondary antibodies in 1% BSA in PBST at room temperature for 1 h. Cell nuclei were stained with 1 µg/mL DAPI during the secondary antibody incubation period.

### Engineering of HepG2-NTCP-C4-HBV-RADARS stable cell line

To generate HepG2-NTCP-C4 stably expressing HBV-RADARS reporter, the reporter segment of HBV-RADARS plasmid was PCR amplified using primers ATCGGAATTCTAGCGTTTAAACTTAAGCTT and TATGGCTGATTATGATCTCTAGTCAGGTTTAAACGGGCCCTCTAG, followed by EcoRI and XbaI restriction digestion before ligating into the pLVX-Puro (Takara) lentiviral vector by T4 DNA ligase (Takara). Pseudotyped lentiviruses were packaged by Lenti-X Packaging Single Shots (VSV-G) (Takara) in Lenti-X 293T cells (Takara). Produced lentiviruses were applied to HepG2-NTCP-C4 cells, followed by puromycin (2 µg/mL) selection for 2 weeks. The puromycin-resistant cells were expanded and designated as HepG2-NTCP-C4-HBV-RADARS.

### Statistical analysis

Data shown in the bar graphs indicate mean ± standard deviation. Data were analyzed using two-way ANOVA analysis for multiple group analysis, or two-tailed Student’s *t-*test for comparing two groups. All bar graphs and statistics were generated by Prism GraphPad 10. The level of significance was set at *P* < 0.05.

## SUPPLEMENTAL MATERIAL

Figure S1 jvi.00922-25-s0001.TIF Sequence alignment between HBV-RADARS sensor and target regions from genotypes A, B, C, and D.

Figure S2 jvi.00922-25-s0002.TIF Activation of HBV-RADARS corresponds to its target HBV RNA levels in HepG2.2.15.

Figure S3 jvi.00922-25-s0003.TIF Binding between HBV-RADARS RNA and target RNA does not significantly induce IFN-β.

Figure S4 jvi.00922-25-s0004.TIF Effects of ADAR1 overexpression on HBV-RADARS-GFP reporter in HBV-infected cells.

Figure S5 jvi.00922-25-s0005.TIF Characterization of HBV-RADARS stable cell line in *de novo* HBV infection.

Supplemental legends jvi.00922-25-s0006.docx Descriptive legends for supplemental material.

Table S1 jvi.00922-25-s0007.xlsx List of primers used in this study.

Table S2 jvi.00922-25-s0008.xlsx List of synthesized DNA sequences used in this study.

ASM does not own the copyrights to Supplemental Material that may be linked to, or accessed through, an article. The authors have granted ASM a non-exclusive, world-wide license to publish the Supplemental Material files. Please contact the corresponding author directly for reuse.
