# Supplementary figures and images for: A hepatitis B virus RNA-sensing and RNA-editing-dependent reporter system
Source: J Virol. 2025 Oct 10;99(11):e00922-25. doi: 10.1128/jvi.00922-25 (PMC12645999; doi:10.1128/jvi.00922-25)

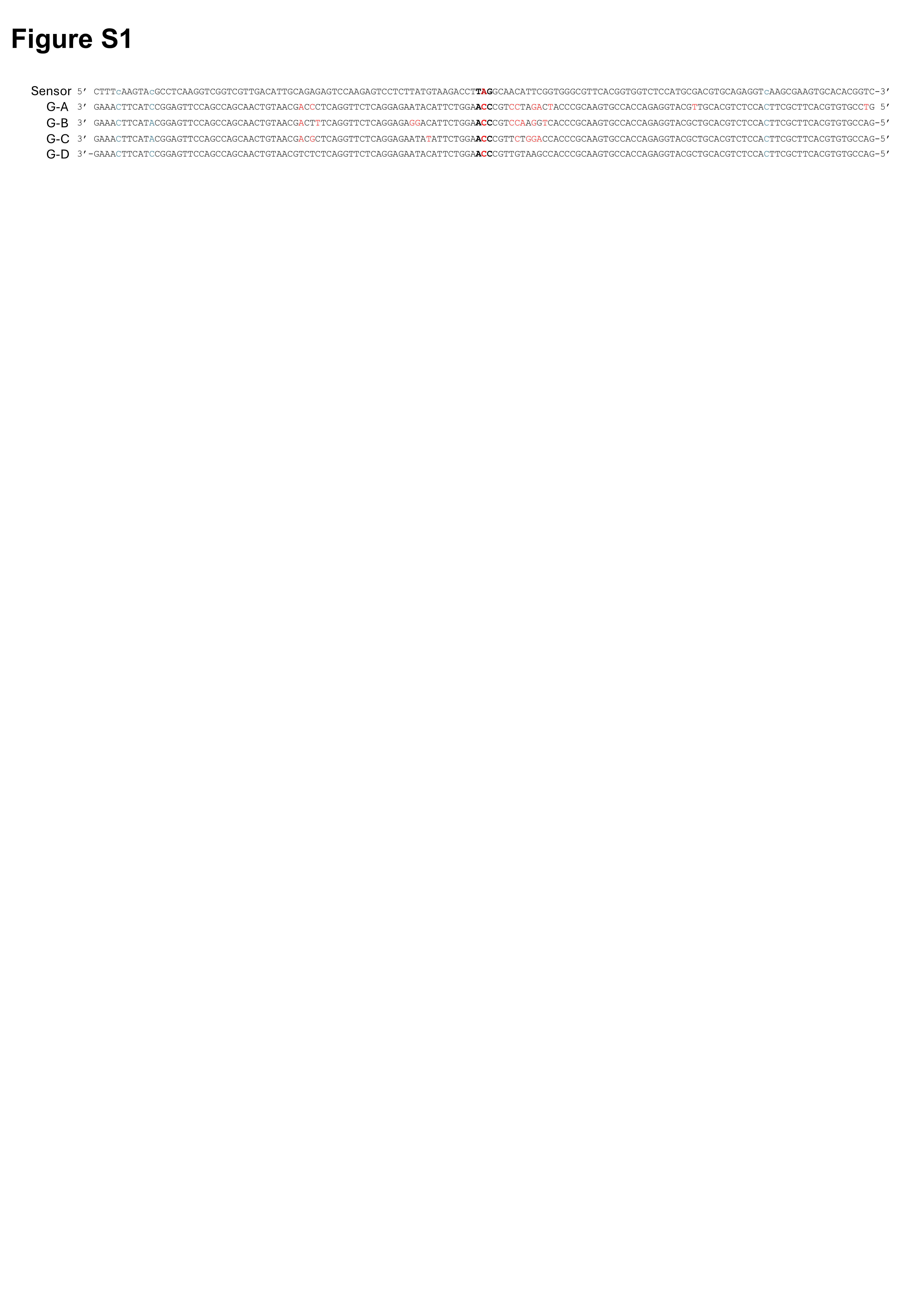

Supplement: Figure S1 — Sequence alignment between HBV-RADARS sensor and target regions from genotypes A, B, C, and D. [file jvi.00922-25-s0001.tif]

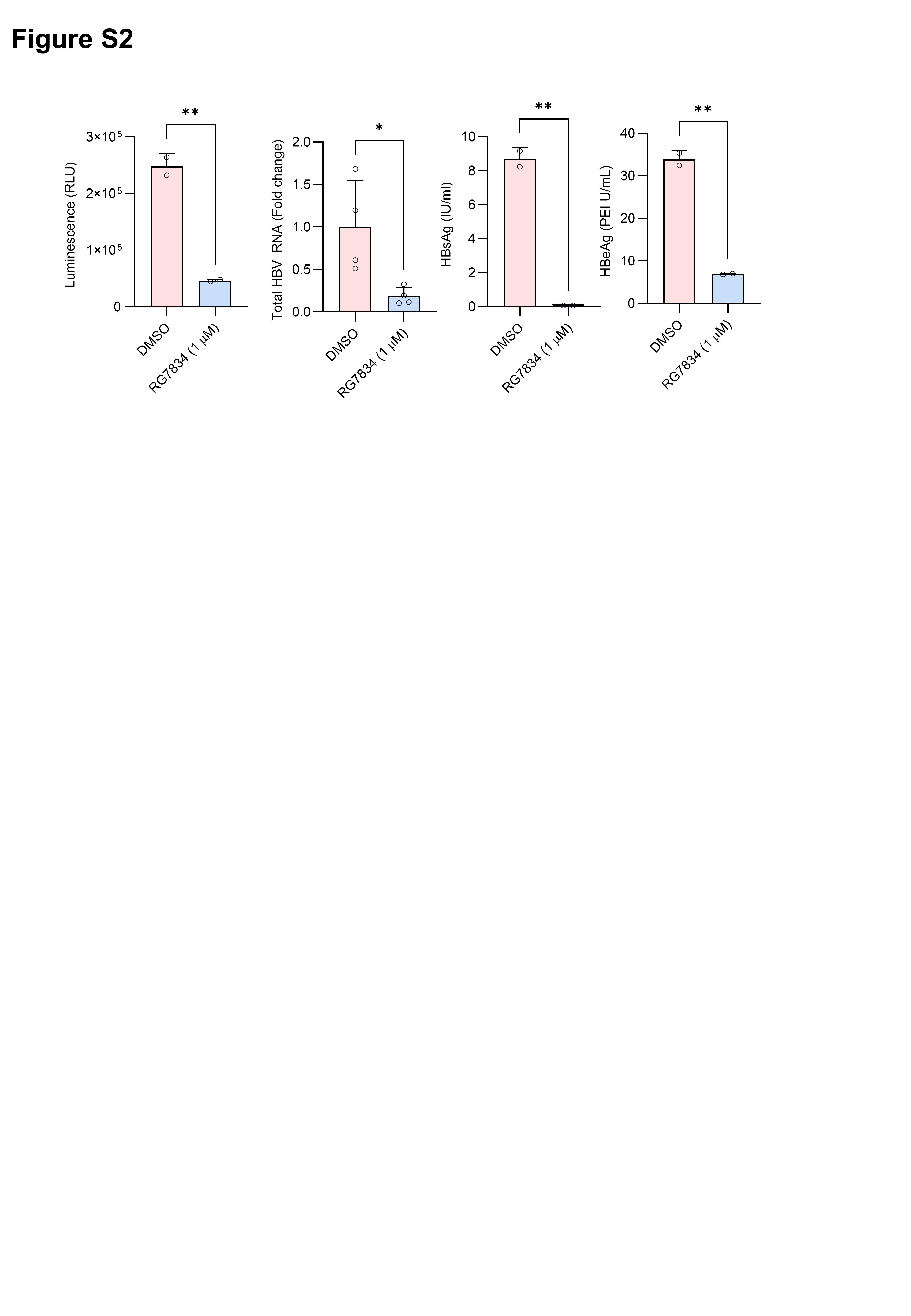

Supplement: Figure S2 — Activation of HBV-RADARS corresponds to its target HBV RNA levels in HepG2.2.15. [file jvi.00922-25-s0002.tif]

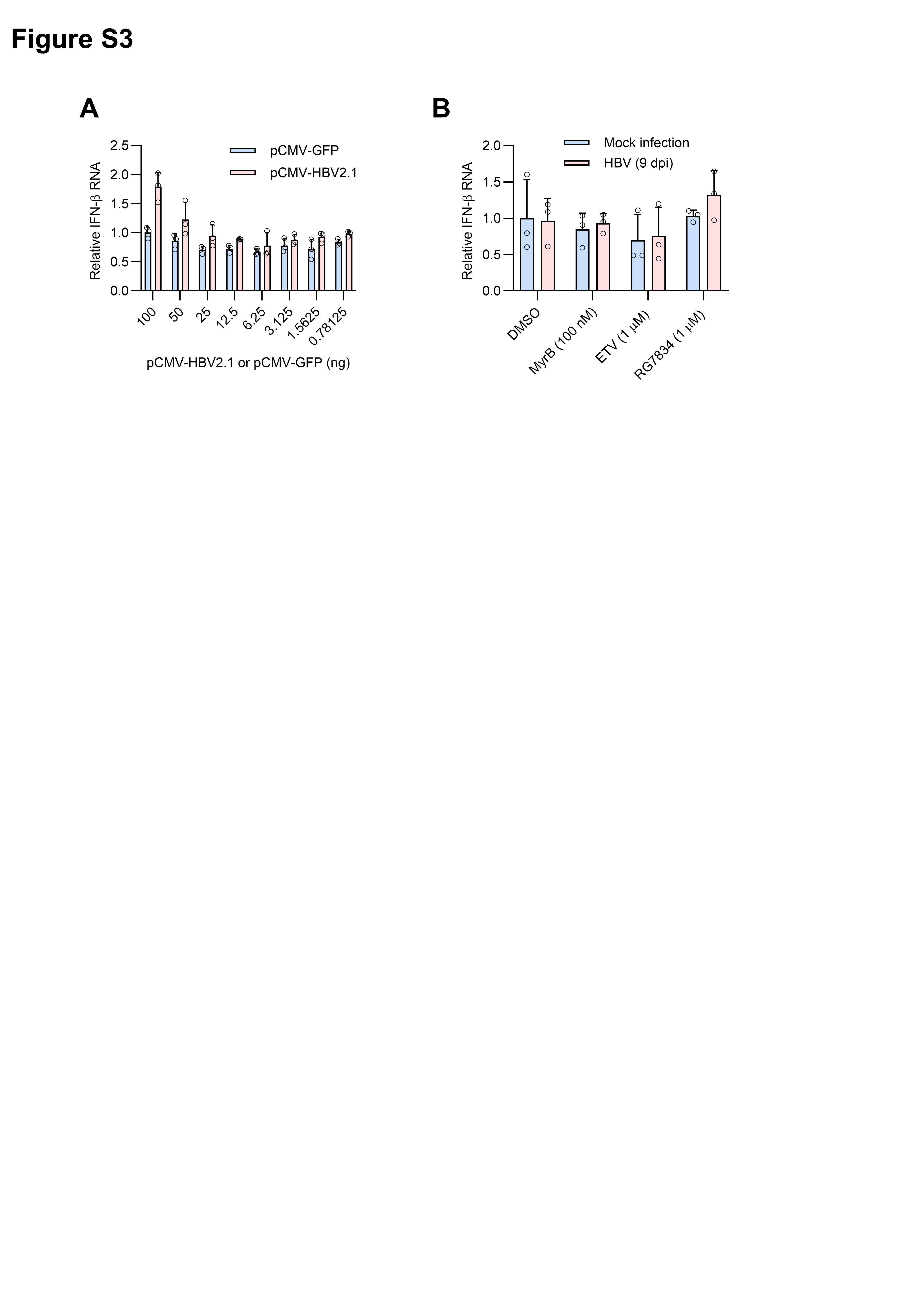

Supplement: Figure S3 — Binding between HBV-RADARS RNA and target RNA does not significantly induce IFN-β. [file jvi.00922-25-s0003.tif]

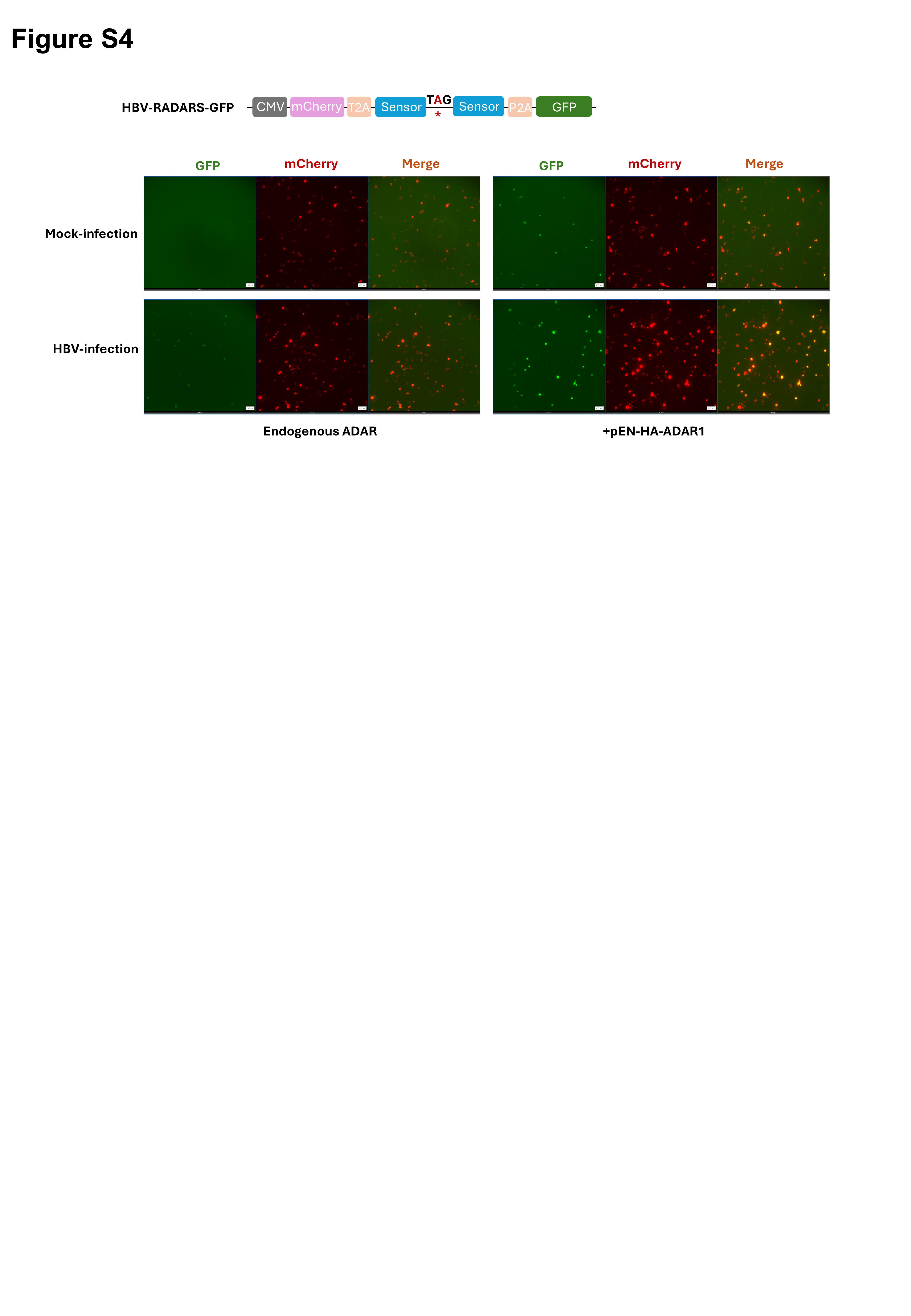

Supplement: Figure S4 — Effects of ADAR1 overexpression on HBV-RADARS-GFP reporter in HBV-infected cells. [file jvi.00922-25-s0004.tif]

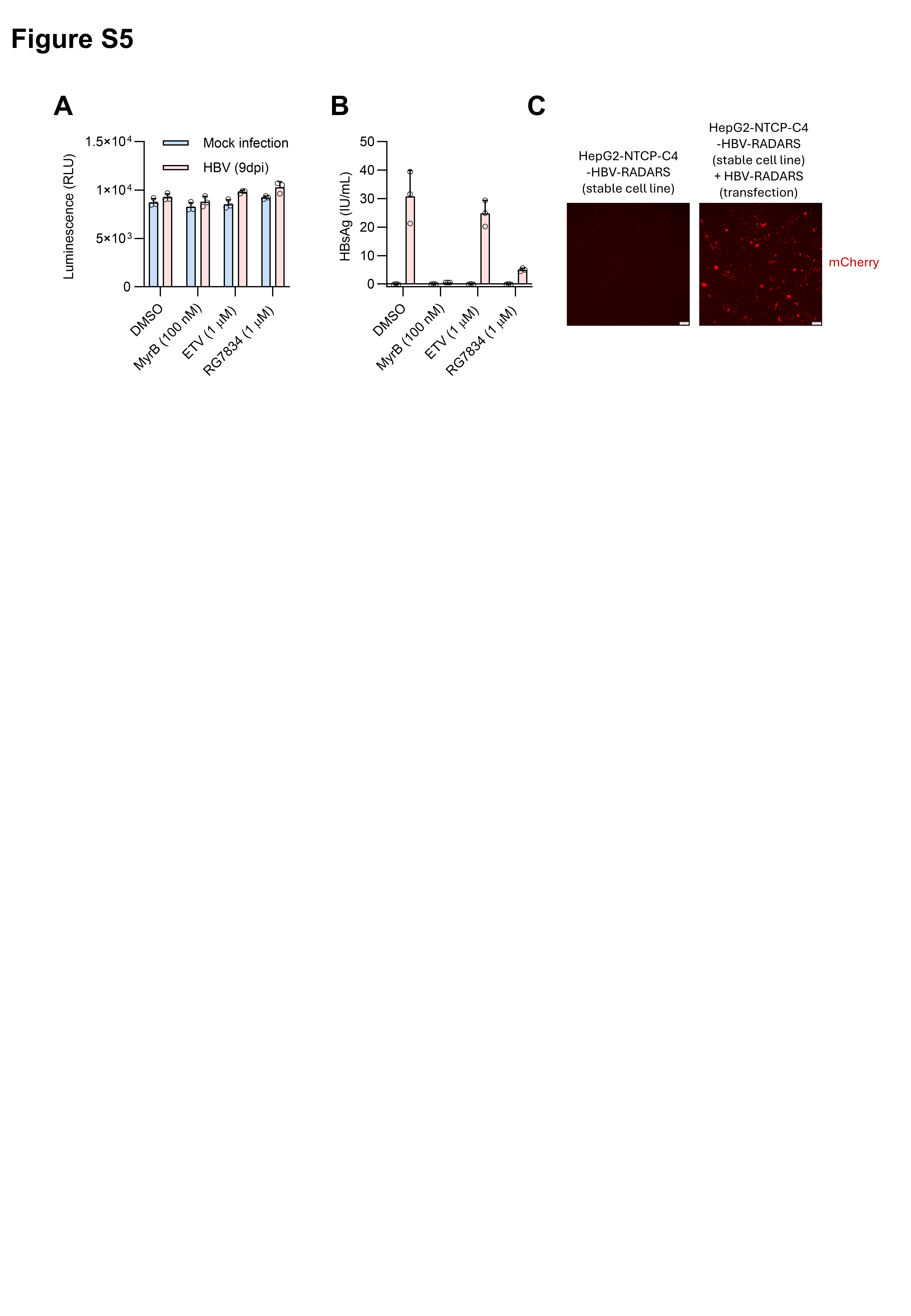

Supplement: Figure S5 — Characterization of HBV-RADARS stable cell line in de novo HBV infection. [file jvi.00922-25-s0005.tif]
