# Supplementary material for: A hepatitis B virus RNA-sensing and RNA-editing-dependent reporter system
Source: J Virol. 2025 Oct 10;99(11):e00922-25. doi: 10.1128/jvi.00922-25 (PMC12645999; doi:10.1128/jvi.00922-25)
Supplement: Supplemental legends — Descriptive legends for supplemental material. [file jvi.00922-25-s0006.docx]

**Supplemental Figures and Tables**

**Fig S1. Sequence alignment between HBV-RADARS sensor and target regions from genotypes A, B, C, and D**. Mismatched sequences between the 141-nt sensor RNA and HBV genotypes A, B, C, and D are shown in red. Nucleotides colored in blue (lower case in HBV-RADARS) specify ones that are purposefully not matched to avoid potential additional in-frame and out-of-frame start or stop codons that may affect HBV-RADARS translation.

**Fig S2. Activation of HBV-RADARS corresponds to its target HBV RNA levels in HepG2.2.15.** HepG2.2.15 cells seeded in 24-well plate were transfected with 200 ng HBV-RADARS reporter plasmid, and then treated with DMSO or 1 µM of RG7834 from day 1 to day 4 post transfection. The Gluc luminescence was measured. Total HBV RNA transcripts were quantified by RT-qPCR with two technical replicates and normalized to cellular β-actin RNA. Data are presented as HBV RNA levels relative to the DMSO-treated group. HBsAg and HBeAg secreted into culture supernatant from day 1 to day 4 were quantified by with the CLIA. Mean ± SD is shown with 2 biological replicates. *, P < 0.05; **, P < 0.01.

**Fig S3. Binding between HBV-RADARS RNA and target RNA does not significantly induce IFN-β.** (**A**) HepG2-NTCP-C4 cells seeded in 48-well plate were transfected with 100 ng fixed amount of HBV-RADARS plasmid, while co-transfected with indicated amounts of pCMV-HBV2.1 or pCMV-GFP for 4 days. Cellular IFN-β RNA transcripts were quantified by RT-qPCR and normalized to cellular β-actin RNA. Data are presented as IFN-β RNA levels relative to that in 100 ng pCMV-GFP transfected group. (**B**) HepG2-NTCP-C4 cells were transfected with HBV-RADARS at 1 day prior to HBV infection, followed by mock-infection or infection with HBV at 5,000 GEs/cell. MyrB treatment indicates addition of MyrB during viral inoculation. One day after viral inoculation, cells were either left untreated (DMSO) or treated with ETV or RG7834 until 9 dpi. Medium was refreshed every other day. Cellular IFN-β RNA transcripts were quantified by RT-qPCR and normalized to cellular β-actin RNA. Data are presented as IFN-β RNA levels relative to that in mock-infected and DMSO-treated group. Mean ± SD is shown with 3 biological replicates.

**Fig S4. Effects of ADAR1 overexpression on HBV-RADARS-GFP reporter in HBV-infected cells.** HepG2-NTCP-C4 cells were transfected with HBV-RADARS-GFP reporter plasmid with or without co-transfecting pEN-HA-ADAR1 plasmid. One day after plasmid transfection, cells were either mock-infected or infected with HBV at 5,000 GEs/cell. GFP and mCherry fluorescent images were taken at 4 dpi.

**Fig S5. Characterization of HBV-RADARS stable cell line in *de novo* HBV infection.** HepG2-NTCP-C4 stably expressing HBV-RADARS through lentiviral transduction were mock infected or infected with HBV at 5,000 genome equivalents per cell (5,000 GEs/cell). MyrB was added during viral inoculation. One day after viral inoculation, cells were either left untreated (DMSO) or treated with ETV or RG7834 until 9 dpi. Medium was refreshed every other day. (**A**) The Gluc luminescence was measured. (**B**) HBsAg secreted into culture supernatant from 7 dpi to 9 dpi were quantified with the CLIA. (**C**) The mCherry fluorescent images for HepG2-NTCP-C4-HBV-RADARS stable cell line with or without additional HBV-RADARS plasmid transfection were taken at 9 dpi. Mean ± SD is shown with 3 biological replicates.

**Table S1. List of primers used in this study.**

**Table S2: List of synthesized DNA sequences used in this study.**
